# Clinical, biochemical and molecular characteristics of Filipino patients with mucopolysaccharidosis type II - Hunter syndrome

**DOI:** 10.1186/s13023-016-0558-0

**Published:** 2017-01-11

**Authors:** Mary Anne D. Chiong, Daffodil M. Canson, Mary Ann R. Abacan, Melissa Mae P. Baluyot, Cynthia P. Cordero, Catherine Lynn T. Silao

**Affiliations:** 1 Institute of Human Genetics, National Institutes of Health, University of the Philippines Manila, 625 Pedro Gil St., Ermita, Manila 1000 Philippines; 2 Department of Pediatrics, University of the Philippines-Philippine General Hospital, Manila, Philippines; 3 Department of Pediatrics, College of Medicine, University of Santo Tomas, Manila, Philippines; 4 Department of Clinical Epidemiology, College of Medicine, University of the Philippines, Manila, Philippines

**Keywords:** Mucopolysaccharidosis type II, Hunter syndrome, Iduronate-2-sulfatase gene, Lysosomal storage disease, Glycosaminoglycans

## Abstract

**Background:**

Mucopolysaccharidosis type II, an X-linked recessive disorder is the most common lysosomal storage disease detected among Filipinos. This is a case series involving 23 male Filipino patients confirmed to have Hunter syndrome. The clinical and biochemical characteristics were obtained and mutation testing of the *IDS* gene was done on the probands and their female relatives.

**Results:**

The mean age of the patients was 11.28 (SD 4.10) years with an average symptom onset at 1.2 (SD 1.4) years. The mean age at biochemical diagnosis was 8 (SD 3.2) years. The early clinical characteristics were developmental delay, joint stiffness, coarse facies, recurrent respiratory tract infections, abdominal distention and hernia. Majority of the patients had joint contractures, severe intellectual disability, error of refraction, hearing loss and valvular regurgitation on subspecialists’ evaluation. The mean GAG concentration was 506.5 mg (SD 191.3)/grams creatinine while the mean plasma iduronate-2-sulfatase activity was 0.86 (SD 0.79) nmol/mg plasma/4 h. Fourteen (14) mutations were found: 6 missense (42.9%), 4 nonsense (28.6%), 2 frameshift (14.3%), 1 exon skipping at the cDNA level (7.1%), and 1 gross insertion (7.1%). Six (6) novel mutations were observed (43%): p.C422F, p.P86Rfs*44, p.Q121*, p.L209Wfs*4, p.T409R, and c.1461_1462insN[710].

**Conclusion:**

The age at diagnosis in this series was much delayed and majority of the patients presented with severe neurologic impairment. The results of the biochemical tests did not contribute to the phenotypic classification of patients. The effects of the mutations were consistent with the severe phenotype seen in the majority of the patients.

## Background

Mucopolysaccharidosis type II (Hunter Syndrome) is an X-linked disorder with an incidence of 0.3–0.71 per 100,000 live births [1]. In the Philippines, there is no reported incidence of Hunter syndrome. Forty two patients have been recorded in the lysosomal storage disease registry of the Institute of Human Genetics-National Institutes of Health, Manila since 1999. The disorder is caused by a deficiency in the lysosomal enzyme iduronate-2-sulfatase (I2S), leading to an accumulation of the glycosaminoglycans dermatan sulfate and heparan sulfate [2]. The *IDS* gene is located in Xq28, spans 24 kb and contains 9 exons. An *IDS*-like pseudogene comprised of copies of exons 2 and 3 and intron 7 is located about 20 kb from the active gene [2].

Patients with the disease are classified as having the severe, intermediate or attenuated forms, depending on the degree of mental retardation present. The severe form appears between 2 and 4 years of age and is characterized by progressive neurologic and somatic involvement. Death usually occurs in the first or second decade of life mostly due to the cardiopulmonary complications. A milder form of Hunter syndrome is characterized by preservation of intelligence and survival into adulthood but with obvious somatic involvement [2]. Patients classified as intermediate usually have mild to moderate learning difficulties and less severe skeletal disease [3].

Analysis of urine glycosaminoglycans (GAGs) can be used to confirm the suspicion of Hunter syndrome. Excess urinary excretion of dermatan sulfate and heparan sulfate is characteristic of Hunter syndrome but not diagnostic as these GAGs can also be elevated in other types of mucopolysaccharidoses. Thus, measurement of iduronate-2-sulfatase enzyme activity is necessary to confirm the diagnosis. Absent or low I2S activity in males is diagnostic of Hunter syndrome but absolute enzyme activity cannot be used to predict the severity of the phenotype [1].

Genetic testing of the iduronate-2-sulfatase gene (*IDS*) may allow prediction of the phenotype. It is also the only reliable way to identify female carriers of the disease which is a critical factor in family planning decisions [4]. Mutations identified in the patients included large alterations and small gene alterations which further confirmed the extreme heterogeneity of *IDS* gene alterations, as more than 350 have been reported to date [5, 6].

This study is the first attempt to characterize the clinical, biochemical and molecular characteristics of Filipino patients with Hunter syndrome and aims to describe the phenotype and genotype aspects of the disease.

## Methods

### Study design and participants

This is a case-series of patients aged 1–21 years old who were diagnosed at the Philippine General Hospital (PGH) or Institute of Human Genetics (IHG) between 1999 and 2015 and listed in the Lysosomal Storage Disease Registry of the Institute of Human Genetics, National Institutes of Health, the only institution in the Philippines providing genetic services. Written informed consent from the parents and/or patients was obtained prior to participation. Patients had a clinical diagnosis of Hunter syndrome which was further confirmed biochemically by demonstrating a high excretion of glycosaminoglycans in the urine and a deficiency in iduronate-2-sulfatase activity in leukocytes. The mothers and other female members of the family of the patients who consented also underwent mutation studies. Pedigree analysis was done in each family and genetic counseling was provided after confirmation of the diagnosis. The study protocol was approved by the institution’s ethical review board (2012-329-01).

### Clinical characteristics

The data on the age at onset of symptoms, age at diagnosis, early clinical signs and symptoms as well as their developmental histories were obtained from the medical records of the Philippine General Hospital. The patients were also asked to come for clinic evaluations where medical specialists assessed the patients’ general appearance and determined any skeletal, ophthalmologic, otorhinolaryngologic, gastrointestinal, cardiovascular, pulmonary and neurologic abnormalities.

Despite the lack of a standardized scoring index of severity for patients with Hunter syndrome, in this series, the severity of the neurologic disease was used to arrive at a particular form of classification. They were classified according to the following by the clinical geneticists taking care of them: severe if the patients had moderate to severe intellectual disability and or neurodegeneration; intermediate if they had mild intellectual disability or learning difficulties; and attenuated if they had no behavioral disturbance or mental retardation regardless of the severity of bone and visceral involvement [3, 5] In terms of intellectual disability, the patients’ adaptive functions were categorized by the developmental pediatricians who attended to them in the clinic based on the DSM 5 (Diagnostic and Statistical Manual for Mental Disorders) criteria for developmental quotient (DQ of 50–70: mild intellectual disability; DQ 35–50: moderate intellectual disability; DQ of 20–35 severe intellectual disability and DQ <20: profound intellectual disability).

### Biochemical studies

All 23 patients in the study had urinary glycosaminoglycans and plasma IDS activity measurements on their leukocytes. These tests were sent to the laboratory of the National Taiwan University Hospital. The concentration of urinary glycosaminoglycans was measured using the Dimethylene Blue assay in relation to urinary creatinine. The results were compared with the established reference ranges per age group of urinary GAGs per grams of creatinine. The plasma IDS activity was measured using the 4-methylumbelliferone (4-MU) fluorometric enzyme assay.

### Mutation studies

The peripheral blood from the patients underwent DNA extraction using the QIAgen QIAamp Blood Midi kit. The coding region of the *IDS* gene was amplified using both previously described and newly designed oligonucleotide primers. Bi-directional Sanger sequencing of nine exon-specific amplicons containing flanking intronic regions was performed using the ABI 3130 Genetic Analyzer [3, 7]. Nested PCR was specifically done for the amplification of exon 3 to avoid co-amplification of a homologous region in the *IDS* pseudogene. Mutation confirmation and heterozygote detection were subsequently performed by sequencing the *IDS* genomic amplicons containing the identified mutation using DNA obtained from possible carriers.

Where PCR amplification of exon 8 using genomic DNA failed, RNA was extracted from 2.5 ml of whole blood using the PreAnalytiX PAXgene Blood RNA kit, then subsequently applied as template in cDNA synthesis using Invitrogen M-MLV Reverse Transcriptase. A forward primer within exon 7 and a reverse primer within exon 9 were designed to detect the exon 8 deletion through gap PCR. Confirmation of the deletion was carried out by sequencing the PCR product.

### Methods of data analysis

The quantitative patient characteristics such as age at diagnosis were summarized by means and standard deviations (SDs). The qualitative characteristics such as general appearance, skeletal abnormalities, and other organ complications were presented as a frequency distribution.

## Results

### Clinical findings

A total of 23 male patients belonging to 21 families participated in the study. The mean age of the patients at the time of the study was 11.28 (SD 4.10) years and the mean age at onset of symptoms reported for 20 patients was 1.2 (SD 1.43) years ranging from as early as the day of birth to as late as 6 years of age. The onset for the other three patients was reported as ‘less than 1 year of age’. Two of these patients had umbilical hernia at birth. The mean age at biochemical diagnosis was 7.6 (SD 3.58) years. The earliest age that the diagnosis was confirmed biochemically was 7 months and the latest was 13.5 years. There were four patients in this study who belonged to two sets of families.

**Fig. 1:**
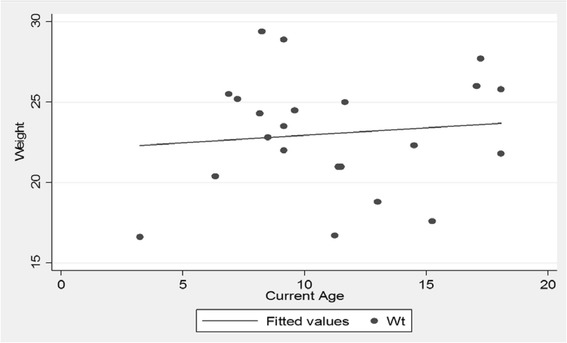
Scatter plot of age and weight. Patients gained weight as they grew older

**Fig. 2:**
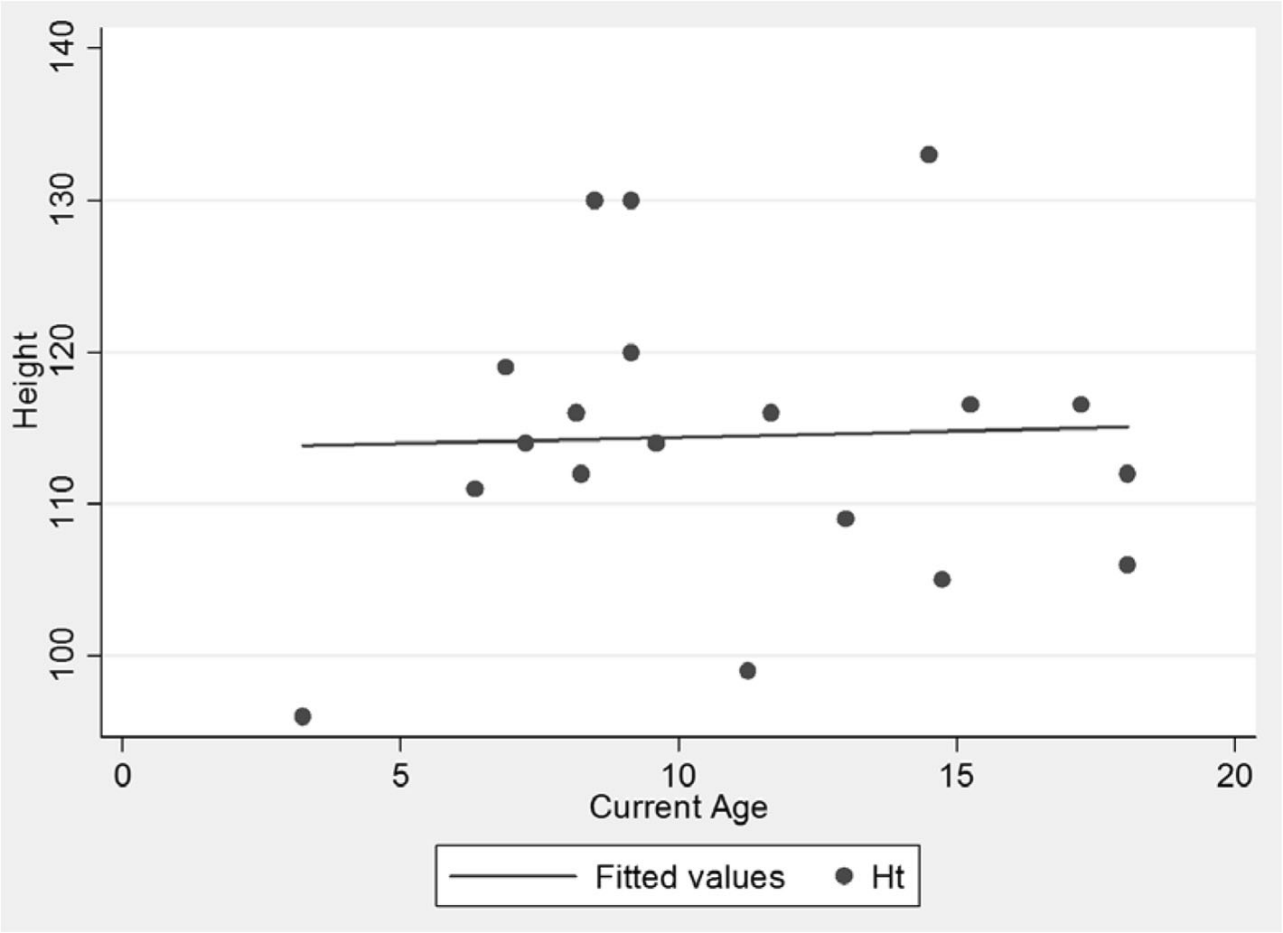
Scatter plot of age and height. Patients’ heights flattened as they grew older

**Fig. 3:**
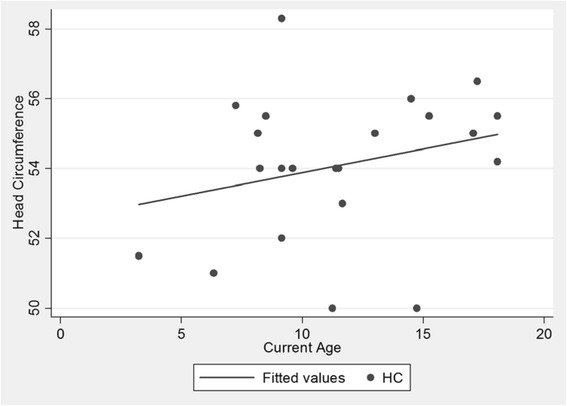
Scatter plot of age and head circumference. Patients’ head circumference grew bigger as they got older

The mean weight at the time of evaluation was 23 (SD 3.6) kg, mean height was 114.5 (SD 9.7) cm and mean head circumference was 54 (SD 2.1) cm. There was a slight increase in weight as the patients grew in age (Fig. 1) but the pattern for height showed a flatter line as they grew older (Fig. 2). The head circumference was increasing as the patient got older (Fig. 3).

**Fig. 4:**
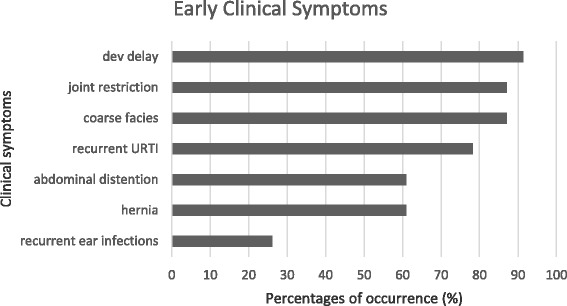
Reported early clinical symptoms among Filipino patients with Hunter syndrome. The most common symptoms were developmental delay, followed by joint stiffness, coarse facies, recurrent upper respiratory tract infections, abdominal distention and hernia and recurrent ear infections

The early clinical characteristics observed by the parents were developmental delay (21/23; 91.3%), followed by joint stiffness and coarse facies (20/23; 87%), recurrent upper respiratory tract infections (18/23; 78.3%), abdominal distention and hernia (14/23; 61%) and recurrent ear infections (6/23; 26%) (Fig. 4). The parents also recalled that their sons were told by physicians during the course of the disease to have hepatosplenomegaly (9/23; 39%), airway obstruction (9/23; 39%), papular rash (6/23; 26%), kyphoscoliosis (4/23; 17%), valvular thickness (4/23; 17%), papilledema (3/23; 13%) and hip dysplasia (1/23; 4%).

**Fig. 5:**
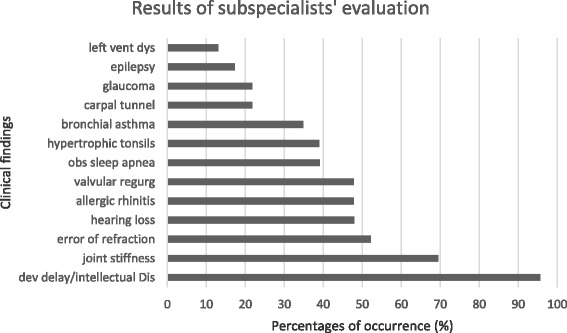
Clinical characteristics of MPS II patients noted during subspecialists’ evaluations. More than half of the patients had intellectual disability, joint stiffness and error of refraction

On subspecialists’ evaluations (Fig. 5), 52% (12/23) presented with severe intellectual disability while 17% (4/23) had moderate intellectual disability. Twenty-one percent (5/23) had mild intellectual disability and 1 patient (4%) was developmentally and intellectually at par with age upon formal developmental assessment at 9 years of age. One patient had global developmental delay at 3 years old but the severity could not be determined yet at the time of evaluation. Based on this neurodevelopmental assessment using the DSM 5 criteria, 16 patients were classified as having the severe phenotype (69.5%), five patients had the intermediate phenotype (21.7%) and only one patient had the attenuated phenotype. The boy who was found to have global developmental delay at 3 years of age could not be classified to either severe or intermediate phenotype yet. Unfortunately, majority of the patients were not completely assessed by brain imaging or electroencephalography. Four patients (17.3%) were diagnosed to have epilepsy but only one patient was able to undergo an electroencephalogram which revealed abnormal epileptiform discharges. One patient also had hydrocephalus on computed tomography scan of the brain (4.3%). Carpal tunnel syndrome was observed in 5 patients (22%), and other symptoms that suggested a possible peripheral neuropathy were seen in two patients (8.7%). One patient was diagnosed to have autism spectrum disorder. Only 11 out of the 23 (48%) patients attended school at the time of evaluation. None of the patients with severe intellectual disability attended school.

Ophthalmologic findings showed error of refraction (12/23; 52%), glaucoma, (5/23; 21.7%), and other eye findings such as Meibomian gland dysfunction and pigmentary retinopathy (5/23;21.7%). Hearing loss was profound in 4/23 (17.4%) patients, moderate to profound in 2 (8.7%), moderate in 1 (4.3%) and unclassified in 4 (17.4%). Only five patients were using hearing aids. Twelve patients had no hearing loss at the time of this study (52.2%).

Hypertrophic tonsils were present in 9/23 patients (39%). Obstructive sleep apnea was seen in nine patients (39%) and another nine were suspected to have this disorder but did not undergo sleep studies yet. Bronchial asthma was seen at 34.8% (8/23) and allergic rhinitis at 47.8% (11/23).

Mild valvular regurgitation which involved the aortic and mitral valves was noted at 47.8% (11/23) and left ventricular dysfunction at 13% (3/23). Left ventricular hypertrophy was seen in two patients (8.7%) and one patient was noted to have valvular thickness of the mitral valve (4.3%).

Joint contractures were seen in 16 out of 23 patients (69.57%). Only two patients had a skeletal survey done which showed dysostosis multiplex (8.7%).

None of the patients ever received enzyme replacement therapy for Hunter syndrome.

### Biochemical findings

The mean GAG concentration for 22 patients was 506.5 mg (SD of 191.3) per grams creatinine. Majority of the patients had GAG between 300 and 800 mg per grams creatinine which were all beyond the average reference values for age across the different age groups (<1 year old: 90.76 mg/grams creatinine; 3–5 years old: 45.16 mg/grams creatinine and >5 years old: 35.74 mg/grams creatinine). The GAG analysis was not done for 1 patient with a severe type of disease.

The mean glycosaminoglycan (GAG) concentration for patients with severe disease was 471.5 mg (SD 174.9) per grams creatinine (range 126.1–778.6 mg/grams creatinine); for those with intermediate type of the disease, the mean was 534.2 mg (SD 86.8) per grams creatinine (range 302.0–692.6) and the patient with attenuated disease had a GAG excretion of 443.7 mg per grams creatinine. The patient with global developmental delay whose phenotype could not be assessed yet had a GAG excretion of 891.6 per grams creatinine. The GAG analysis that was performed did not include the amount of the specific type of glycosaminoglycan excreted.

**Fig. 6:**
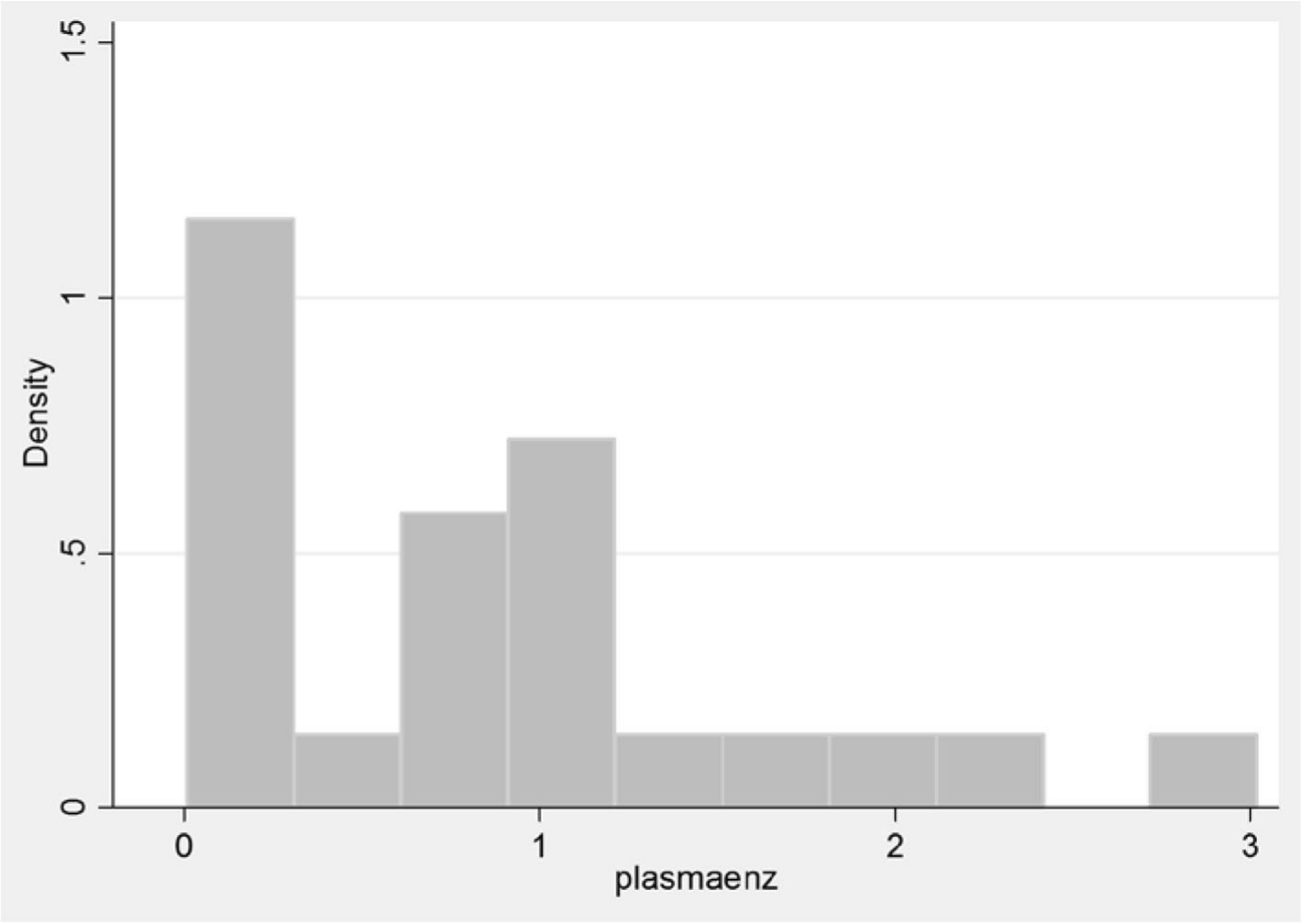
Distribution of Iduronate-2-sulfatase (in nmol/mg/plasma/4 h) levels among 23 patients with Hunter syndrome. The distribution of plasma iduronate 2 sulfatase activity was skewed indicating that regardless of the phenotype, most had levels of less than 1 nmol/mg/plasma/4 h

The plasma enzyme assay for iduronate-2-sulfatase activity for those with a severe type of the disease had a mean of 1.09 and a SD of 0.82 nmol/mg plasma/4 h. It ranged from 0.01 to 3.02. For the intermediate type, the mean was 0.45 (SD 0.36) nmol/mg plasma/4 h. It ranged from 0.1 to 0.91. The patient with the attenuated type had an activity of 0.01 nmol/mg plasma/4 h. The one who had global developmental delay also had an enzyme level of 0.01 nmol/mg plasma/4 h). Overall, the mean plasma iduronate-2-sulfatase activity was 0.86 (SD 0.79) nmol/mg plasma/4 h. As seen in Fig. 6, the distribution of plasma iduronate 2 sulfatase activity was skewed indicating that regardless of the phenotype, most had levels of less than 1 nmol/mg/plasma/4 h.

### Molecular characteristics

#### Studies on probands

Fourteen (14) different mutations were found in this study among 23 patients belonging to 21 families (Table 1). Six (6) were missense mutations (42.9%), 4 were nonsense (28.6%), 2 were frameshifts (14.3%), 1 was exon skipping at the cDNA level (7.1%), and 1 was a gross insertion (7.1%). Most of the mutations were found in exon 3 (36%). Previously reported mutations p.Q75*, p.P86L, p.R88H, p.W109*, p. R172*, p.R468Q, and p.R468W were found. Six (6) novel mutations were observed (43%). The novel mutation p.C422F was found in 3 patients who are siblings. The other novel mutations found in unrelated patients were p.P86Rfs*44, p.Q121*, p.L209Wfs*4, p.T409R, and c.1461_1462insN[710]. The insertion of about 710 bp in exon 9 is yet to be fully characterized. The length of the insertion was only estimated by agarose gel electrophoresis (data not shown). The mutations p. Q75*, p. P86L, and p.Q121* were found to have occurred *de novo*.

**Table 1:** Mutation studies of 23 patients with MPS II

Patient No.	Mutation	Consequence	Location	Phenotype	Status	Reference
P1	c.1265G > T	p.C422F	Exon 9	Intermediate	novel	-
P2	c.1265G > T	p.C422F	Exon 9	Intermediate	novel	-
P3	c.1265G > T	p.C422F	Exon 9	Intermediate	novel	-
P4	c.(1007 + 1_1008-1)_ (1180 + 1_1181-1)del	EX8del (cDNA level)	Exon 8	Severe	for further characterization at genomic DNA level	-
P5	c.326G > A	p.W109*	Exon 3	Intermediate	published	Brusius-Facchin et al., 2014 [20]
P6	none detected	-	-	Severe	-	-
P7	c.1403G > A	p.R468Q	Exon 9	Severe	published	Whitley et al., 1993 [24]
P8	c.1403G > A	p.R468Q	Exon 9	Severe	published	Whitley et al., 1993 [24]
P9	c.326G > A	p.W109*	Exon 3	Severe	published	Brusius-Facchin et al., 2014 [20]
P10	c.1403G > A	p.R468Q	Exon 9	Severe	published	Whitley et al., 1993 [24]
P11	none detected	-	-	Severe	-	-
P12	c.[626delT; 629A > G]	p.L209Wfs*4	Exon 5	Severe	novel	-
P13	c.1402C > T	p.R468W	Exon 9	Severe	published	Crotty et al., 1992 [23]
P14	c.223C > T	p.Q75*	Exon 2	Severe	published, *de novo*	Kato et al., 2005 [25]
P15	c.1461_1462insN[710]	-	Exon 9	Severe	novel, for further characterization	-
P16	none detected	-	-	Severe	-	-
P17	c.263G > A	p.R88H	Exon 3	Severe	published	Rathmann et al., 1996 [22]
P18	c.(254_257)delC	p.P86Rfs*44	Exon 3	Severe	novel	-
P19	none detected	-	-	Intermediate	-	-
P20	c.1226C > G	p.T409R	Exon 9	Attenuated	novel	-
P21	c.361C > T	p.Q121*	Exon 3	Severe	novel, *de novo*	-
P22	c.257C > T	p.P86L	Exon 3	Global developmental delay	published, *de novo*	Popowska et al., 1995 [21]
P23	c.514C > T	p.R172*	Exon 5	Severe	published	Flomen et al., 1992 [27]

Exon 8 skipping was identified in the cDNA of one patient. Complete deletion of exon 8 in the *IDS* transcript had been previously reported. One case was caused by a 3254-bp deletion in genomic DNA from intron 7 to intron 8 with an insertion of 20 bp [8]. Another case was also caused by an extensive deletion of about 3 kb but with a longer insertion of 157 bp [9]. However, it could not be ascertained whether either of these was the same as the mutation detected in this study since the deletion breakpoints in the genomic DNA in our patient have not yet been defined.

Most of the patients with moderate to severe cognitive impairment or those belonging to the severe phenotype had the following mutations: p.Q75*, p.P86Rfs*44, p.R88H, p.W109*, p.Q121*, p.R172*, p.L209Wfs*4, p.R468Q, p.R468W, Ex8del, and c.1461_1462insN[710]. Three patients presenting with severe phenotype were not found to have any mutation in the exons examined. Four patients presenting with mild learning difficulties (intermediate phenotype) had the mutations p.W109* (one patient) and p.C422F (three patients). One patient with mild learning difficulties did not have any mutations in the exons examined. The mutation p.W109* was found in two patients who are first cousins, but one of them presented with a severe phenotype and died at 12 years of age due to respiratory failure while the other has only mild learning difficulties at 15 years of age. The mutation p.T409R was found in the lone patient with attenuated phenotype who had normal cognition at 9 years of age.

#### Family studies

**Fig. 7:**
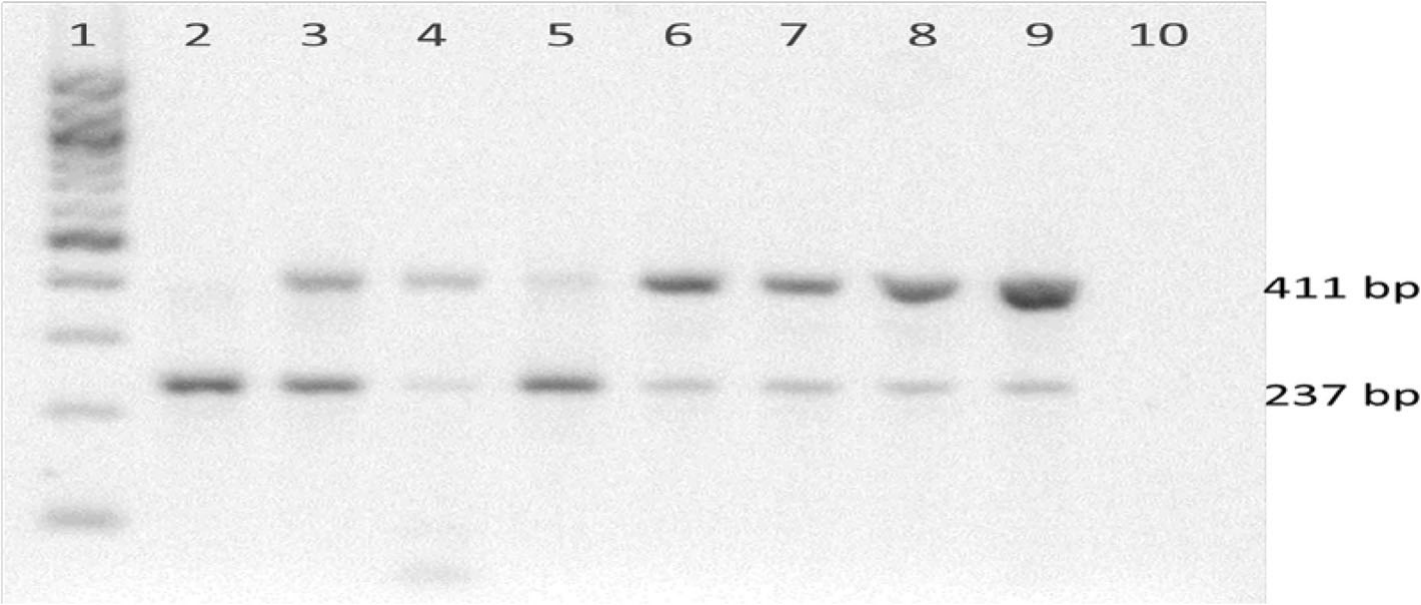
Gel image of carrier testing for exon 8 skipping at cDNA level through gap PCR; 1:100-bp DNA ladder, 2: patient, 3: patient’s mother, 4–8: other female family members, 9: healthy female control, 10: negative control

Carrier testing done on 40 mothers and other female members of each family showed that 20/40 (50%) were carriers. Eleven of the 15 mothers (73%) tested were found to be carriers. Three mothers whose children were found to carry the missense mutations p.Q75*, p.P86L and p.Q121* did not have the said mutations. Carrier testing for exon 8 skipping showed inconclusive results because the same shortened transcript represented by a 237-bp amplicon was also present in the control sample from a healthy female, making the identification of true carriers of genomic deletion of exon 8 uncertain (Fig. 7). However, the band from the healthy control was noticeably fainter than the band from the patient’s mother who is most probably a carrier of the genomic deletion. The 237-bp amplicon isolated from the healthy control had identical sequence with that of the patient’s. To check for a contamination problem, RNA from another healthy female was extracted then reverse transcribed into cDNA separately, but the same gap PCR result persisted. Whether a variant *IDS* transcript lacking exon 8 is actually produced in small quantities in healthy individuals remains to be clarified and further investigated.

## Discussion

This is the first study done on the clinical, biochemical and molecular characteristics of Filipino patients with Hunter syndrome. Our data showed that the onset of disease was early at a mean age of 1 year but the confirmation of diagnosis was done at a mean age of 7 years. Therefore, it took an average of 6 years before the children were diagnosed correctly and managed appropriately. Despite the clinical features present among the patients, late recognition and confirmation of the diagnosis was a usual problem encountered in this series. In the initial report from the Hunter Outcome Survey (HOS) [10], the average age at diagnosis was 4 years of age. The delay in the diagnosis in our series may be due to the lack of awareness among physicians to recognize such constellation of features in one specific syndrome. A common pitfall when examining the patient with undiagnosed Hunter syndrome is a failure to link the many, seemingly unrelated signs and symptoms experienced by the patient into a single syndrome [11]. Another reason could be the lack of facilities dedicated for patients with rare diseases in the Philippines. Similar to a study done in Brazil [10], the delay in the diagnosis of patients with Hunter syndrome or any rare metabolic disease in general could be mainly due to the structure of the health system in the Philippines. Being at the bottom of the government’s priority list, there are very few health clinics and specialists that can comprehensively assess these types of patients.

The phenotypic expression of Hunter syndrome spans a wide spectrum of clinical severity. If neurologic involvement is the main basis of classification for the severity of the disorder, it can be deduced that most of the patients included in this study were skewed towards the severe end of the spectrum as majority of them presented with moderate to severe intellectual disability. In a worldwide Hunter Outcome Survey survey which started in 2005, 84% of the 263 subjects enrolled in the study showed neurologic involvement [12], verifying the assumption that the severe phenotype may be more prevalent than the attenuated phenotype [13].

The most common clinical characteristics reported in this series were compatible with what has already been reported in the literature. Apart from developmental delay and intellectual disability seen in majority of our patients, most also had coarse facies, joint restriction, respiratory problems, hepatosplenomegaly, and abdominal hernia. The most prevalent clinical characteristics observed in the HOS was facial dysmorphism followed by respiratory tract abnormalities such as otitis media, nasal obstruction, and enlarged tongue and adenoids. Hepatosplenomegaly, abdominal hernia and joint stiffness were likewise prevalent [12]. Similarly, in the clinical study done on 77 patients with Mucopolysaccharidosis type II in Brazil, joint contractures, macrocephaly, coarsened facial features and increased abdominal volume/hepatosplenomegaly were the most frequently reported early clinical manifestations [10].

On subspecialists’ evaluation, the most common neurologic symptom apart from developmental delay/intellectual disability was epilepsy. Behavioral abnormalities were not frequently reported except for one patient diagnosed to have autism spectrum disorder.

Error of refraction seen in this series is, indeed, a common finding in patients with Hunter syndrome [10]. Glaucoma although not frequently reported in the literature was present in 21% of the cases. In an unpublished local study done on 15 patients with Hunter syndrome (Roa et al., 2012, unpublished data) all were found to have error of refraction, the most common being hyperopia. Other findings included strabismus, tessellated retinas, pigmentary retinopathy, and large cup-to-disc ratios. None had corneal clouding. Being one of the most common systems affected in patients with Hunter syndrome, early detection and management of eye problems can have a profound impact on the quality of life especially in terms of their independence in day to day activities thus, full ophthalmologic evaluation should be regularly instituted.

Bronchial asthma was found in 35% of our patients and allergic rhinitis was noted in 50% of them. These two conditions may be due to the reactive airway disease that happens when there is mucosal swelling, GAG accumulation, and inflammation in the nasal passages or bronchi of patients with Hunter syndrome as what is also similarly seen in other types of mucopolysaccharidosis [14]. The high incidence of airway obstruction and sleep apnea found in this case series should alert the physicians in suspecting and recognizing a possible mucopolysaccharidosis when such symptoms are seen.

Cardiovascular involvement was seen in 80% of the patients, with mild valvular regurgitation being the most common. This data is congruent with the reports from HOS wherein the prevalence of cardiac involvement is high among these patients and that valvular disease is the most common finding [12, 14]. Given this, physicians should aggressively assess the cardiac function of these children and evaluate them for other reported cardiac findings such as hypertension, arrhythmia and congestive heart failure as these pose a significant cause of morbidity and mortality [15].

In this study, there seemed to be no relation between the severity of the cognitive impairment and the concentration of the glycosaminoglycan excretion and plasma iduronate-2-sulfatase assay. It was noted that the patients with the intermediate disease even had higher GAG excretion and lower plasma iduronate-2-sulfatase activities compared to those with the severe phenotype. The levels of GAG and iduronate-2-sulfatase in the patient with the attenuated phenotype also fit in the ranges found in the group with the severe phenotype. In a study done in Korea [16], plasma iduronate-2-sulfatase activity in the patients with the severe type had significantly lower values than in the attenuated type of the disease. It was not possible to corroborate this finding in this series as most of the patients presented with neurologic impairments. The levels of heparan sulfate in the urine which were previously found to correlate with the severity of Hunter syndrome [17] was not specifically determined in this cohort of patients.

The 14 mutations found in the 23 patients reflect the genetic heterogeneity seen in Hunter syndrome. The severe phenotypes found in the patients who presented with the following mutations, p.P86L, p.R88H, p.R468Q, and p. R468W, are in agreement with those reported in literature [3, 18–24]. Specifically, the above mutations have also been found in the Asian population such as in Chinese and Japanese patients [4, 25, 26] presenting with the severe phenotype. The published nonsense mutations, p.Q75*, p.W109*, and p.R172*, were found in our patients with severe phenotypes and were in agreement with previous literature reports among Caucasian and Asian patients with Hunter syndrome in terms of their effects on phenotype [19, 20, 27–29]. Mutations introducing premature translation termination codons trigger nonsense-mediated decay, which prevents the synthesis of an abnormal protein, and have commonly been classified as severe mutations [4]. However, one of the patients carrying the mutation p.W109* presented with an intermediate phenotype compared with his cousin who carried the same mutation and manifested with a severe phenotype. This could be explained by the imperfect clinical correlation between patients with the same mutation [3, 27].

With regard to the pathogenicity of the novel mutations, the frameshift mutations caused by single-base deletions, p.P86Rfs*44 and p.L209Wfs*4, are both predicted to introduce a premature stop codon downstream that could also trigger nonsense-mediated decay, which is consistent with the severe phenotype. The gross insertion in exon 9,c.1461_1462insN[710], probably led to the destabilization of the tertiary structure of the protein resulting to a severe phenotype.

The novel missense mutations, p.C422F and p.T409R, although probably damaging to protein function based on the PolyPhen-2prediction algorithm (available at http://genetics.bwh.harvard.edu/pph2/bgi.shtml, accessed on 31 July 2015), with scores 0.994 and 1.000, respectively, gave rise to less severe phenotypes. The p.C422F mutation was found in three siblings with mild learning difficulties, while p.T409R was found in the single patient with no cognitive dysfunction.

## Conclusions

The clinical characteristics of Mucopolysaccharidosis type II in this case series were in agreement with what has been reported in the literature except that the age at confirmation of diagnosis is much delayed despite earlier onset of symptoms. Majority of the patients presented with neurologic impairment with different grades of severity. The biochemical tests showed no relation with the consequent phenotype among the patients. The molecular analysis showed eight previously reported and six novel mutations, the effects of which were consistent with the severe phenotype seen in the majority of the patients.

Our findings emphasize the need for early recognition of Hunter syndrome among physicians and that there should be a heightened suspicion among them for the characteristic signs and symptoms so that delay in diagnosis can be avoided. Improvement in the referral system for expert clinical evaluation as well as suitable biochemical and molecular diagnosis will aid in the provision of multidisciplinary care and appropriate genetic counseling for the families. Likewise, in order to maintain a better quality of life for these patients, a comprehensive disability assessment on the activities of daily living (ADLs) should also be initiated so that they can get proper help in their specific areas of difficulties and evaluate improvements and deteriorations in important domains over time. Availability and accessibility of enzyme replacement therapy and other novel drugs will additionally be greatly beneficial to these patients and multi-subspecialty management remains essential. The passage of the National Rare Disease Act in March 2016 (Republic Act No. 10747, 2016) which specifies the formulation of a comprehensive and sustainable health system for orphan or rare disorders will hopefully address the pitfalls in the diagnosis and treatment of our patients with Hunter syndrome in the near future.

## Abbreviations

GAG: Glycosaminoglycans
HOS: Hunter outcome survey
I2S: Iduronate-2-sulfatase
IDS: Iduronate-2-sulfatase gene
IHG: Institute of Human Genetics
MPS II: Mucopolysaccharidosis type II
PGH: Philippine General Hospital
